# Characterization of one sheep border disease virus in China

**DOI:** 10.1186/s12985-014-0217-9

**Published:** 2015-02-08

**Authors:** Li Mao, Xia Liu, Wenliang Li, Leilei Yang, Wenwen Zhang, Jieyuan Jiang

**Affiliations:** Institute of Veterinary Medicine, Jiangsu Academy of Agricultural Sciences; Key Laboratory of Veterinary Biological Engineering and Technology, Ministry of Agriculture; National Center for Engineering Research of Veterinary Bio-products, Nanjing, 210014 China; Jiangsu Co-innovation Center for Prevention and Control of Important Animal Infectious Diseases and Zoonoses, Yangzhou, 225009 China

**Keywords:** BDV, Complete genome sequence, Phylogenetic analysis, Experimental infection

## Abstract

**Background:**

Border disease virus (BDV) causes border disease (BD) affecting mainly sheep and goats worldwide. BDV in goat herds suffering diarrhea was recently reported in China, however, infection in sheep was undetermined. Here, BDV infections of sheep herds in Jiangsu, China were screened; a BDV strain was isolated and identified from the sheep flocks in China. The genomic characteristics and pathogenesis of this new isolate were studied.

**Results:**

In 2012, samples from 160 animals in 5 regions of Jiangsu province of China were screened for the presence of BDV genomic RNA and antibody by RT-PCR and ELISA, respectively. 44.4% of the sera were detected positively, and one slowly grown sheep was analyzed to be pestivirus RNA positive and antibody-negative. The sheep kept virus positive and antibody negative in the next 6 months of whole fattening period, and was defined as persistent infection (PI). The virus was isolated in MDBK cells without cytopathic effect (CPE) and named as JSLS12-01. Near-full-length genome sequenced was 12,227 nucleotides (nt). Phylogenetic analysis based on 5'-UTR and N^pro^ fragments showed that the strain belonged to genotype 3, and shared varied homology with the other 3 BDV strains previously isolated from Chinese goats. The genome sequence of JSLS12-01 also had the highest homology with genotype BDV-3 (the strain Gifhorn). Experimental infections of sheep had mild clinical signs as depression and short-period mild fever (5 days). Viremia was detected in 1–7 days post-infection (dpi), and seroconversion began after 14 dpi.

**Conclusions:**

This study reported the genomic and pathogenesis characterizations of one sheep BDV strain, which confirmed the occurrence of BDV infection in Chinese sheep. This sheep derived BDV strain was classified as BDV-3, together with the goat derived strains in China. These results might be helpful for further understanding of BDV infection in China and useful for prevention and control of BDV infections in the future.

## Background

*Pestivirus* is a genus within family *Flaviviridae*, comprises four recognized species, namely border disease virus (BDV) of small ruminants, bovine viral diarrhea viruses 1 and 2 (BVDV-1, BVDV-2) of cattle and classical swine fever virus (CSFV) of pigs, respectively [1]. Recently, new pestivirus species, such as Giraffe virus, Pronghorn virus, Bungowannah virus and HoBi-like virus have been discovered [2,3].

BDV infects small ruminants (sheep and goats) and causes border disease worldwide. Several BDV strains have been proved to infect pigs and cattle under experimental or nature conditions [4,5]. BD shares some similar characteristics with BVDV infections in cattle and goats, however, with more pronounced emphasis on a wide range of productive diseases, such as abortions, stillbirths or mummified fetuses, barren ewes, malformations and the birth of weak lambs, which leads to a considerable economic effect. The affected lambs show abnormal body conformation and “hairy-shaker” syndrome named due to the hairy fleeces and tremors of the suffered lambs [6]. Small weak lambs can be persistently infected (PI) and the virus is wide spread in all organs in infected animals. However, BDV infections in pregnant goats result exclusively in abortions and malformations in fetuses and neonates, and PI goats are rarely found out via placenta [7]. An experiment infection was carried out in pregnant ewes with BDV-4, besides a high number of stillbirths up to 32%, significantly reduced bodyweight of lambs was also observed [8]. Although severe clinical outbreaks of BD are unusual, several epizootics have been reported in sheep and goats [9-11].

The pestivirus genome consists of a positive single-stranded RNA approximately 12.3 kb in length, encoding a single open reading frame (ORF) flanked by 5'- and 3'- untranslated regions (UTR). The genome codes 4 structural proteins, the capsid (C) and three envelope proteins (E^rns^, E1 and E2), plus seven or eight non-structural proteins [12,13]. Except the 5'-UTR region, the N^pro^ and E2 genes have also been used for genetic classification of new virus isolates [14,15]. Based on recent reports, BDV isolates have been divided into seven genotypes at least, and widely distributed in different countries, such as many European countries, Australia, New Zealand, Canada, the United States, India, Turkey, and Japan [7,16]. In 2012, BDV infections were first confirmed in several goat herds suffering serious diarrhea, and three BDV strains were isolated in China [11]. This was the first confirmed evidence of BDV genotype 3 circulations in Chinese goats, In order to further investigate the epidemic information of BDV in goats and sheep in the same regions of China, BDV sero-epidemiological survey was carried out in our lab. One BDV strain named JSLS12-01 was isolated from one slow-grown sheep. And the genomic characteristics and pathogenesis of the isolate was determined. The data might be more supplement for BDV epidemiology, pathogenicity as well as its relationship with clinical diseases in China.

## Results

### Antibody and viral detection

One hundred and sixty sera from sheep and goats in five regions of Jiangsu, China were collected and detected by BDV ELISA kits (SVANOVA). The positive rate was 44.4% (71/160), with 46.3% (63/136) of goats and 33.3% (8/24) of sheep, respectively. The positive rates varied from 16.7% (2/12) to 85.7% (12/14) with the flocks (Table 1). Sero-prevalence was at least 50% in two sheep herds and one goat herd from two regions of Jiangsu (Table 1).

**Table 1:** **Results of the diagnostic tests performed with samples using ELISA and RT-PCR**

**Farm**	**Regions**	**Goats/sheep**	**ELISA**	**RT-PCR**
1	Suining, Xuzhou	Goats	85.7%(12/14)	1/14
2	Suining, Xuzhou	sheep	16.7%(2/12)	0/12
3	Siyang, Suqian	Goats	36%(9/25)	1/25
4	Rudong, Nantong	Goats	34.2%(13/38)	NT
5	Rudong, Nantong	sheep	50%(4/8)	0/8
6	Haian, Nantong	Goats	49.2%(29/59)	NT
7	Lishui, Nanjing	sheep	50%(2/4)	1/4

NT, Sample not tested.

In the sheep herds, an antibody-negative sheep (2 month old) from Nanjing, Jiangsu was detected positive by RT-PCR, which showed the very bright bands of 290 bp and 225 bp amplified by panpesti generic primers and BDV specific primers PBD1/PBD2, respectively. The RT-PCR products were purified and sequenced, which shared high homology with BDV strains by BLAST analysis. The RT-PCR results also showed two samples from goats were tested positive, 5'-UTR sequences were compared to sequences from GenBank, and shared 100% homology with JS12-04 strain reported previously.

### Virus isolation

The BDV RNA-positive serum was cultured and passaged in MDBK cells. After the third passage, BDV cultures have been positively detected by RT-PCR and confirmed with sequencing. However, no CPE was observed during the process of passage. The new BDV isolate was named as JSLS12-01.

### Subsequent clinical observations and serological analysis

Four sampling sheep including the BDV positive were continued to rear for clinical investigation subsequently, with the same feed and management conditions. Compared to the BDV negative sheep, the infected lamb was thinner, weaker, and poorer growth, and the body weight was about 20% less at the end of fattening period (6 month long). However, no other clinical signs were observed. The four sheep were tested once 4 weeks apart, and BDV RNA was positively detected by RT-PCR and the BDV specific antibody was negative for the original BDV positive animal in the whole grown period.

### Complete genome sequencing and phylogenetic analysis

**Figure 1:**
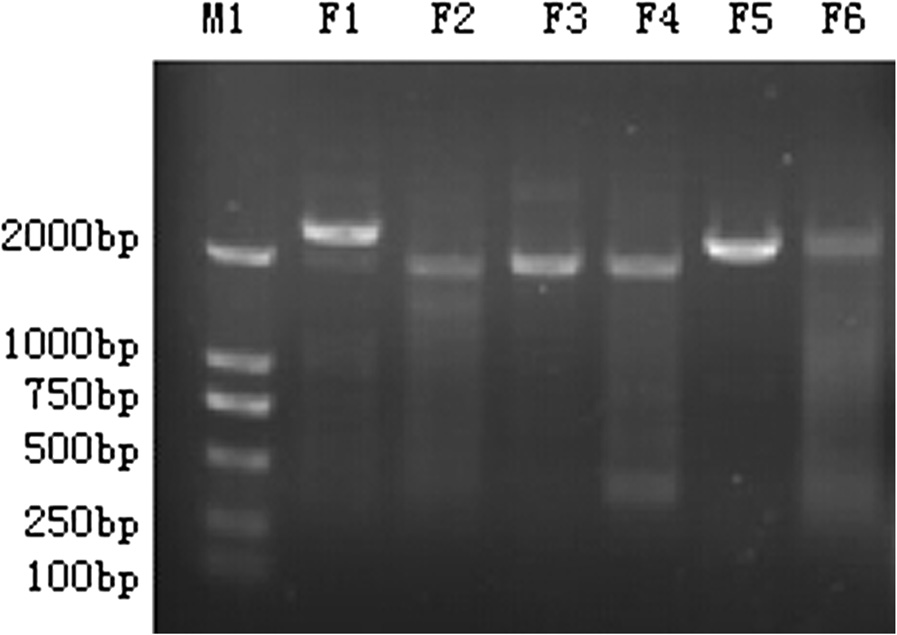
**Six overlapping fragments covering the complete genome of JSLS12-01 (F1 ~ F6).** F1-F6 were six overlapping RT-PCR products covering the virus genome, the size of each fragment was matched sizes in the Table 1. M1: DM2000 marker.

**Figure 2:**
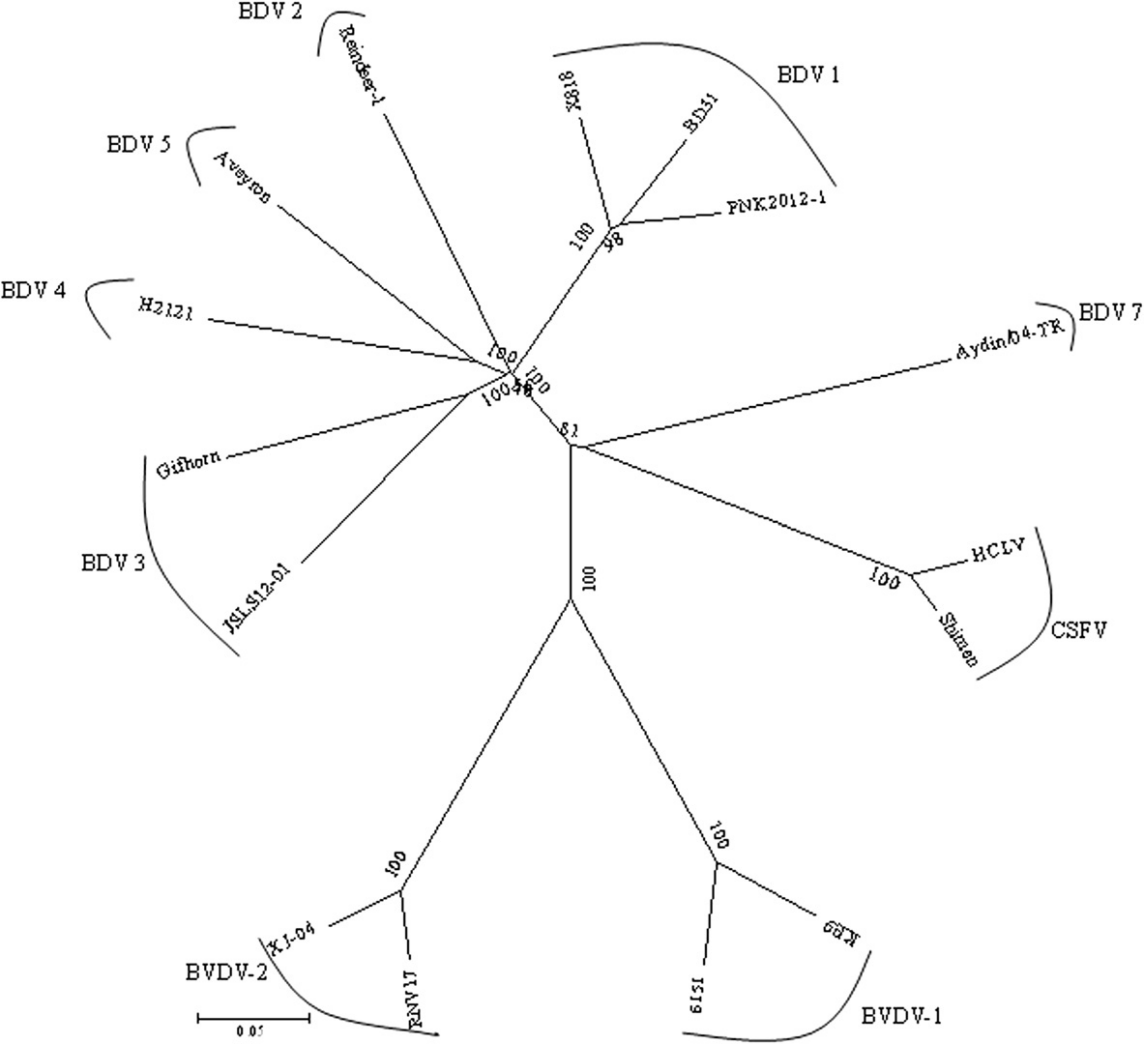
**Phylogenetic analysis based on the complete coding sequence of BDV strains using the Neighbour-joining method.** The numbers close to the major nodes indicate the bootstrap values (in %; 1000 replicates). Bar: number of substitutions per site.

RT-PCR products (Figure 1) were purified and cloned to pJET1.2 vectors for sequencing. The near full genome sequence of BDV JSLS12-01 strain was obtained and deposited in GenBank under accession number KC963426 and reported recently [17], the obtained genome of JSLS12-01 was 12, 227 nucleotides (nt) in length. The isolate shared 80.3% nucleotide homology and 89.9% amino acid homology with goat derived strain Gifhorn (a prototype of BDV-3), respectively (Table 2). It shared 72.2-77.6% homology with other BDV genotypes strains available in GenBank (Table 2). The homology with CSFV and BVDV strains were about 71% and 67%, respectively (Table 2). The whole genome sequence based phylogenetic analysis indicated that JSLS12-01 was classified into the same branch with Gifhorn with 100% bootstrap value, and matched up with the result of the Blast analysis. The isolate clearly differed from other BDV strains and other pestivirus species (CSFVs or BVDVs) (Figure 2).

**Table 2:** **The homology of complete coding gene and protein sequence between JSLS12-01 and partial BDV strains**

**Virus strain**	**GenBank accession number**	**Pestivirus species**	**JSLS12-01**	
			**Homology of complete coding gene (%)**	**Homology of protein (%)**
FNK2012-1	AB897785	BDV-1	77.5	86.4
BD31	U70263	BDV-1	77.0	85.6
X818	AF037405	BDV-1	76.9	85.5
Reindeer-1	AF144618	BDV-2	77.3	86.5
Gifhorn	KF925348	BDV-3	80.3	89.9
H2121	GU270877	BDV-4	77.5	87.2
Aveyron	KF918753	BDV-5	77.6	87.0
Aydin/04-TR	JX428945	BDV-7	72.2	80.1
HCLV	AF091507	CSFV	71.0	78.5
Shimen	AF092448	CSFV	71.2	79.0
KE9	EF101530	BVDV-1	67.0	72.0
6151	JN380083	BVDV-1	67.2	72.6
RNV17	JN380090	BVDV-2	67.4	72.3
XJ-04	FJ527854	BVDV-2	66.9	71.7

**Figure 3:**
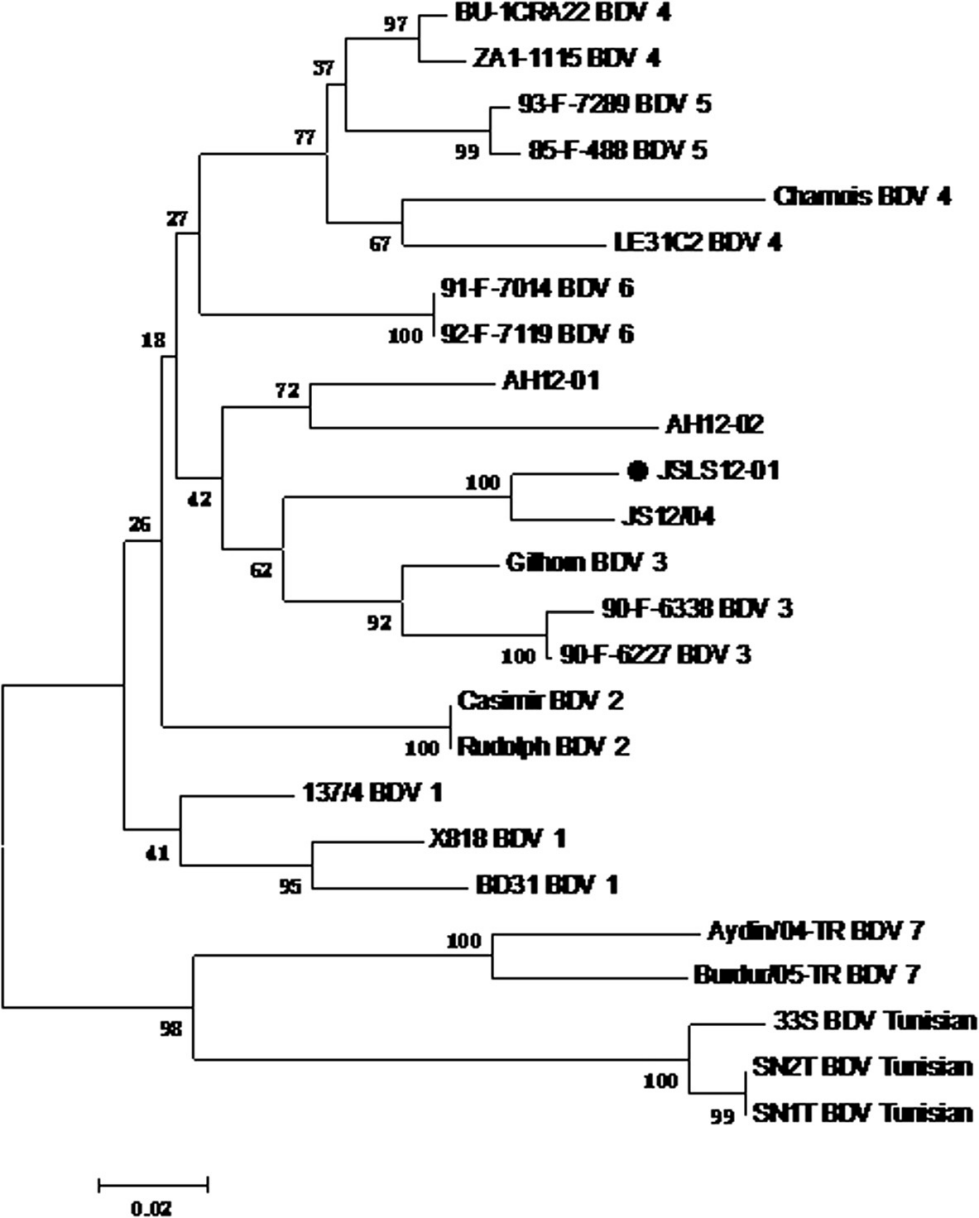
**Neighbour-joining phylogenetic tree constructed using 225 nt 5’-UTR fragments of the pestivirus sequences found in this study and from the GenBank.** Representatives of BDV sequences have been described in Li et al. [11]. The numbers close to the major nodes indicate the bootstrap values (in %; 1000 replicates). Bar: number of substitutions per site.

5'-UTR and N^pro^ sequences of the virus were aligned with the corresponding sequences of BDV reference strains and the evolutionary relationship between these isolates was estimated by phylogenetic analysis to further characterize the JSLS12-01 isolate. The phylogenetic tree based on 5'-UTR region (225 bp) showed that the JSLS12-01 strain was grouped with BDV-3 respective strains (Figure 3). Comparison of 5'-UTR sequences revealed that BDV JSLS12-01 shared nucleotide identities of 78.6%, 83.5% and 94.4% with BDV-3 Chinese strains AH12-02, AH12-01 and JS12-04, respectively. The virus was 87.7%, 87.4% and 89.0% homology with other BDV-3 strains including Gifhorn from Spanish goats, 90-F-6338 and 90-F-6227 from sheep of France, respectively (Table 3); and also 82.5% to 85.7% with BDV-1; 84.1% with BDV-2; 76.4% to 85.1% with BDV-4; 84.2% to 85.0% with BDV-5; 86.7% with BDV- 6; 71.5% to 72.9% with BDV-7; and 70.5% to 72.6% with BDV Tunisian; respectively (Table 3). The highest identity of nucleotide sequence was 94.4% with the strain JS12-04 isolated in 2012 from Chinese goats (Table 3). JSLS12-01 shared the highest homology (73.2% to 80.7%) with other BDV-3 members on the N^pro^ sequences (Table 3). And based on the phylogenetic tree of the N^pro^ sequences also grouped the strain JSLS12-01 into BDV-3 (data not shown).

**Table 3:** **The homology of 5’-UTR and N** ^**pro**^ **genes between JSLS12-01 and partial BDV strains**

**Virus strain**	**Genotype**	**JSLS12-01**	
		**Homology of 5’-UTR gene (%)**	**Homology of N** ^**pro**^ **gene (%)**
X818	BDV-1	82.5	64.1
BD31	BDV-1	82.6	63.7
137/4	BDV-1	85.7	66.3
Casimir	BDV-2	84.1	-
Rudolph	BDV-2	84.1	-
Gifhorn	BDV 3	87.7	73.2
AH12-01	BDV-3	83.5	75.8
AH12-02	BDV-3	78.6	73.2
JS12-04	BDV-3	94.4	80.7
90-F-6338	BDV-3	87.4	75.0
90-F-6227	BDV-3	89.0	74.6
BU-1CRA22	BDV-4	85.1	64.4
ZA1-1115	BDV-4	83.2	-
Chamois	BDV-4	76.4	-
LE31C2	BDV-4	79.3	-
85-F-488	BDV-5	84.2	70.1
93-F-7289	BDV-5	85.0	70.5
91-F-7014	BDV-6	86.7	68.1
92-F-7119	BDV-6	86.7	68.5
Burdur/05-TR	BDV-7	72.9	54.7
Aydin/04-TR	BDV-7	71.5	55.7
SN1T	BDV Tunisian	72.6	53.7
33S	BDV Tunisian	70.5	53.7
SN2T	BDV Tunisian	71.3	53.9

-: The sequence could not be obtained in GenBank.

### Pathogenicity of BDV JSLS12-01

**Figure 4:**
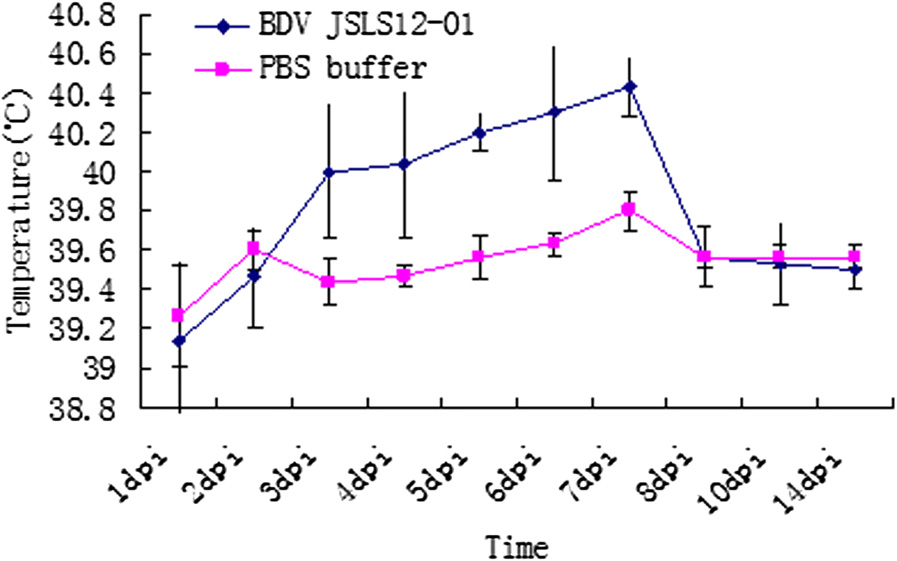
**Body temperature observation of the experimental sheep.** The body temperature of BDV JSLS12-01 infected sheep group and control group were measured at the same time during the first 14 days.

**Figure 5:**
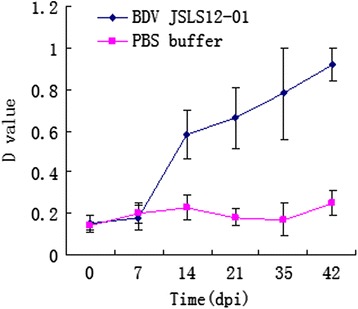
**Antibody test of the experimental sheep.** Serum samples of 0, 7, 14, 21, 35 and 42 dpi were tested for BDV specific antibodies using ELISA kit (SVANOVA).

All 3 lambs (45 day old) infected with BDV JSLS12-01 cell cultures showed only moderated depression without other clinical signs. The infected animals developed high rectal temperatures (40.0-41.0°C), with peak temperatures appearing on 3–7 day-post-infection (dpi) and returned to the normal level on 8 dpi, while the control sheep kept normal during the same period (Figure 4). RT-PCR and virus isolation revealed that the virus could lead to viremia in the infected animals at 1-7dpi. The infected animals began seroconverted on 14 dpi and kept to increase in the following period (Figure 5).

## Discussion

Goats are major rearing small ruminants, and the numbers of sheep and rearing sheep farms are relative a few in Jiangsu and Anhui provinces of eastern China. BDV had been identified in Chinese diseased goats [11]. In the present study, BDV sero-epidemiological survey was carried out for goat and sheep by BDV ELISA kit and RT-PCR. Serum samples from seven flocks (4 of goats and 3 of sheep) in five regions were tested positive by ELISA (Table 1), which indicated the ubiquity of BDV infection in the small ruminant flocks. Four sheep including one was BDV RNA positive with RT-PCR were kept for the next 6 month rearing. The sheep was BDV Ab negative and viremia for the observation period; and identified as one PI animal according to the reports [7]. The PI sheep mainly showed poor-growing were agreement with BDV-4 virus infections [8]; however, classical clinical signs of BD such as paralysis and an abnormal fleece known as ‘hairy shaker’ were not appeared. In addition, the previous goat derived BDVs were identified from herds with diarrhea [11]. These uncharacteristic signs emphasized the difficulty of BD diagnosis based on clinical signs and the requirement for routine and accurate laboratory tests.

Phylogenetic segregations of pestiviruses into individual species and subgroups are only identified by the branching order of the phylogenetic tree [18]. The genetic diversity of BDV is greater than that of other pestivirus species. According to the molecular diversity, BDV have been divided to seven groups at least [7,19,20]. The near full sequence of the JSLS12-01 had a closer relationship with BDV-3 strains at gene level comparing with other pestivirus reference strains (BDV-1,BDV-2, BDV-3, BDV-4,BDV-5 and BDV-7). And the strain should be one new BDV-3 strain with a lot of nucleotide or amino acid variation to the respective BDV strains (Figure 2, Table 2). The isolate JSLS12-01 was the highest homology with the strain JS12-04 on the 5'-UTR and N^pro^ gene compositions, and the two viruses were isolated from sheep or goats in different regions of Jiangsu province, however, the virus JSLS12-01 had much lesser identity with the other two viruses AH12-01 and AH12-02 on the gene levels (Table 3 and Figure 3), and the late two were from Anhui province (neighborhood each other). It indicated the epidemic BDV strains in China might have complex endemic situations, and have existed for a relative long period. The BDVs in China have mutated a lot, and may form different sub-branches, such as the two strains JS12-04 and JSLS12-01 isolated in Jiangsu province formed one cluster, and the strains AH12-01 and AH12-02 from Anhui were much closed each other (Figure 3).

In addition, based on sequence alignment and phylogenetic analysis, the 4 Chinese BDV strains showed the high diversity of on the 5'-UTR compositions (Table 3) even though all of them were in BDV-3 genotype (Figure 3) [11]. The strain JSLS12-01 was only 83.5% and 78.6% homology with the strains AH12-01 and AH12-02 on 5'-UTR gene (Table 3), respectively. Furthermore, it was also found that JSLS12-01 had a very high homology of 5'-UTR sequences with other genotype viruses, such as 85.1%, 86.7% and 85% with the strains BU-1CRA22 (BDV-4), 92-F-7119 (BDV-6) and 93-F-7289 (BDV-5) (Table 3), respectively. It was true that the homology of BDV strains in the same genotype might be much less than the viruses being other genotypes. And it appeared that the BDV genotyping based on the 5'-UTR sequences could be still consummated.

Animal experiment was performed upon conventionally rearing sheep to evaluate the pathogenicity of the new BDV strain. JSLS12-01 could induce mild clinical diseases; the infected animals showed viremia at 1–7 dpi and disappeared after 7 dpi, during the same period, mild short-term pyrexia was observed (Figure 4). In addition, the infected animals seroconverted from 14 dpi (Figure 5). No other classical BD symptom was observed. The clinical symptoms of natural infected sheep mainly showed slow-growing and persisted infection. Symptoms of natural and experimental infection were very diverse, this might be related to the immune state and age of the virus infected animals and so on. To date, experimental infections of BDV have not been performed in China, the results would help to understand the pathogenicity of Chinese BDV strains in small ruminants and further systemic studies will be done in the future.

## Conclusions

In conclusion, BDV infections of sheep were demonstrated in China, and the BDV JSLS12-01 strain was clustered in BDV-3 subgroups, the predominant genotype in China. The BDV infections of goats and sheep appeared with various clinical signs and epidemic information. More studies should be carried out to make sure of the virulence and biologic characteristics of BDV.

## Methods

### Ethics statement

This study was performed in strict accordance with the guidelines of Jiangsu Province Animal Regulations (Government Decree No 45). The protocol was approved by the Committee on the Ethics of Animal Experiments of the Institute of Veterinary Medicine, Jiangsu Academy of Agricultural Sciences (JAAS No 20100604).

### Clinical sampling and observation

Serum samples were collected from 3 young sheep farms and 4 goat farms (about 2 months old) from 5 different regions in Jiangsu province of China (Table 1). A total of 24 sera from sheep and 136 sera from goats were collected. The sheep tested for BDV positive were continuously carried out for subsequent clinical observations and serological analysis in next 6 months (the fattening period) after the first sampling.

### ELISA

BDV antibody detection was performed on serum samples of goats and sheep using a commercially available kit (SVANOVA BDV-Ab kit, SVANOVA Biotech, Uppsala, Sweden) according to the manufacturer’s instructions.

### Virus isolation

Virus isolation was carried out on the positive samples of pestivirus 5'-UTR RT-PCR as reports [16] and briefly introduced as following: the serum samples were centrifuged at 12,000 rpm for 20 min at 4°C and filtered through 0.22 μm filter, and subsequently inoculated onto confluent monolayers of Madin-Darby bovine kidney (MDBK) cells (obtained from China Institute of Veterinary Drugs Control) to culture for 96 hours with 1% FCS DMEM at 37°C and 5% CO_2_ conditions. The original cells and FCS were proven to be free of pestivirus antigen and antibodies. The isolation of BDV was checked by RT-PCR as next described using Panpesti generic primers and BDV specific primers PBD1/PBD2 [21,22].

### RT-PCR detection and complete genomic sequence analysis

Sera from sheep and goats were tested for the presence of pestivirus by RT-PCR method. To detect the BDV virus, total RNA was extracted from serum samples and cell cultures by using the TRizol reagent (Invitrogen) according to the manufacturer’s instruction. RT-PCR was carried out in a 50 μL reaction mixture containing 1× RT-PCR buffer (TAKARA, Bio, Inc.), 20 pM of each primer (Panpesti generic primers and BDV specific primers PBD1/PBD2 targeting 5'-UTR, with expected product sizes of 290 bp and 225 bp, respectively) [21,22], 2 U of one-step Enzyme Mix (TAKARA, Bio, Inc.) and 4 μL of RNA. The reaction was run in a thermocycler (Mjmini, BIO-RAD) according to the following program: reverse transcription at 50°C for 30 min, denaturation at 95°C for 5 min, 35 cycles at 94°C for 30 s, 54°C for 30 s and 72°C for 45 s, terminated with a final extension of 10 min at 72°C.

For the genomic long RNA RT-PCR, the extension time was 1 kb for 1 min. Amplification products were detected by electrophoresis in 1.2% agarose gels. Positive RT-PCR fragments were purified (Axygen), cloned to pJET1.2 vector (Thermo), and then transformed to *E. coli* DH5α. Positive clones, as confirmed by PCR and enzyme digestion, were sequenced. Three positive clones of each RT-PCR fragment were sequenced using the appropriate PCR primers for correct check.

Briefly, six pairs of primers were designed to amplify the 6 overlapping fragments covering the virus genome, and summarized as Table 4. The retrieved sequences were edited and assembled with SeqManTM program version 5.03 of the DNASTAR package to obtain the complete sequence of this new BDV strain.

**Table 4:** **The primer sequence of the complete genome sequence**

**Amplified fragments**	**primer**	**Primer sequences(5'- > 3')**	**Location (bp)**	**Fragment size**
F1	BVDV-F	GCCATGCCCTTAGTAGGACTAGC	1 ~ 23	2200 bp
	BDV-2300R	TATCAGGAAGGCTGTTGTCGA	2255 ~ 2273	
F2	BDV-2240 F	TTGGTGGCCATACGAGACAAC	2144 ~ 2164	1900 bp
	BDV-4140R	TGTCAAGATGAAGAATAGGGG	4023 ~ 4043	
F3	BDV-4010 F	AAGCAGTGGCTACAATCCGTG	3912 ~ 3932	1950 bp
	BDV-5960R	CATCTCTCCAATCCTCAGGTT	5865 ~ 5885	
F4	BDV-5940 F	GCAGAAGCACCCTAGCATAGC	5840 ~ 5860	2000 bp
	BDV-7940R	TATGACTACGCTCTCCAGCCG	7929 ~ 7945	
F5	BDV-7885 F	GCCTTACGCATCTCAAGCCCTC	7787 ~ 7808	2200 bp
	BDV-10110R	TGCCTCGTATGGGTGTATTTTC	10001 ~ 10022	
F6	BDV-10010 F	CAGAGCATATGGTGTCAGCATATCAG	9907 ~ 9932	2300 bp
	BDV-12326R	GGGGCTGTTAGGGTTTTTCCTTAATCC	12201 ~ 12227	

### Phylogenetic analysis

The complete coding sequence of the virus was aligned with some represented BDV, BVDV 1, BVDV 2 and CSFV strain genome sequences. The 5'-UTR and N^pro^ sequences were analyzed with sequences of BDV reference strains using 1.83 and MEGA 4.0.2, the 225 bp 5'-UTR fragments (PBD1/PBD2 product) and 487 bp N^pro^ gene (corresponding to 394-880 bp of Gifhorn genome) sequences were used for analysis, respectively. Phylogenetic analysis was carried out using the neighbor-joining (NJ) method using 1000 replicates for determination the bootstrap values.

### Experimental infection

Six one-month-old healthy sheep were tested negative for pestivirus (BDV and BVDV) infections by commercial ELISA kit (BDV: SVANOVA and BVDV: BIO-X) and RT-PCR mentioned above. They were further confirmed to be free of micoplasma infections by PCR. The sheep were randomly divided into two groups, with 3 animals in each group. Sheep of the experimental group was infected by intramuscular injection with 10^5^ TCID_50_ of BDV JSLS12-01 cell cultures, while the sheep in control group were inoculated with PBS buffer. All animals were monitored daily for clinical signs including depression, nasal discharge, diarrhea, coughing and rectal temperature. Serum samples were collected at day −2 to 0 prior to infection and 1, 3, 5, 7, 14, 21, 28, 35 and 42 dpi. Serum samples of days 1, 3, 5, 7, 14 and 21 were tested for viremia by RT-PCR described above. And the procedures to isolate BDV from the sera were described above. Serum samples of days 0, 7, 14, 21, 35 and 42 were tested for BDV specific antibodies using commercial ELISA kit (SVANOVA).
